# A Bayesian Framework for False Belief Reasoning in Children: A Rational Integration of Theory-Theory and Simulation Theory

**DOI:** 10.3389/fpsyg.2016.02019

**Published:** 2016-12-27

**Authors:** Nobuhiko Asakura, Toshio Inui

**Affiliations:** Department of Psychology, Otemon Gakuin UniversityOsaka, Japan

**Keywords:** false belief, theory-theory, simulation theory, Bayesian network, internal model

## Abstract

Two apparently contrasting theories have been proposed to account for the development of children's theory of mind (ToM): theory-theory and simulation theory. We present a Bayesian framework that rationally integrates both theories for false belief reasoning. This framework exploits two internal models for predicting the belief states of others: one of self and one of others. These internal models are responsible for simulation-based and theory-based reasoning, respectively. The framework further takes into account empirical studies of a developmental ToM scale (e.g., Wellman and Liu, 2004): developmental progressions of various mental state understandings leading up to false belief understanding. By representing the internal models and their interactions as a causal Bayesian network, we formalize the model of children's false belief reasoning as probabilistic computations on the Bayesian network. This model probabilistically weighs and combines the two internal models and predicts children's false belief ability as a multiplicative effect of their early-developed abilities to understand the mental concepts of diverse beliefs and knowledge access. Specifically, the model predicts that children's proportion of correct responses on a false belief task can be closely approximated as the product of their proportions correct on the diverse belief and knowledge access tasks. To validate this prediction, we illustrate that our model provides good fits to a variety of ToM scale data for preschool children. We discuss the implications and extensions of our model for a deeper understanding of developmental progressions of children's ToM abilities.

## 1. Introduction

Inferring and understanding other people's mental states such as desires, beliefs, and intentions is crucial for our successful social interactions. This ability has been referred to as having a “theory of mind” (ToM; Premack and Woodruff, 1978). For decades, ToM development in childhood has been the subject of intensive research; much of the research has focused on children's false belief understanding. Two false belief tasks are widely used for assessing children's ToM: unexpected-contents (Perner et al., 1987) and change-of-location (Wimmer and Perner, 1983) tasks. In the unexpected-contents task, children are shown a familiar container that holds something unexpected inside when it is opened. They are then asked about what an agent will think is inside the container when she has never seen it opened. In the change-of-location task, children are shown a situation in which an agent places an object at one location, leaves the scene, and then her antagonist moves it to another location while she is gone. They are then asked about where the agent will look for the object after she returns. Correct answers for these tasks require children to appreciate that an agent can have a false belief that contradicts the reality with which they are faced. Hence, success on false belief tasks is taken as indicating that ToM has become mature enough to function as an inference engine for reasoning about other people's beliefs, as distinct from one's own.

Many studies have revealed that children come to understand other people's false beliefs at around 4 or 5 years of age; in addition, such a developmental transition appears to occur gradually (e.g., Wellman et al., 2001, for a review). Two main theories have been proposed for explaining the process of ToM development: theory-theory (Gopnik and Wellman, 1992, 1994) and simulation theory (Gordon, 1986; Gallese and Goldman, 1998). Theory-theory assumes that ToM ability rests on a set of rules, or literally theories, about how the minds of others work. It thus claims that children learn and become able to use such theories to predict and explain others' mental states and their behavior. In contrast, simulation theory argues that ToM ability does not require theorizing the minds of others. Instead, it claims that children come to use their own minds as a simulation model to mimic and understand the minds of others.

These theories have long been regarded as contrasting conceptualizations of ToM. In recent years, however, a number of researchers have advocated hybrid theories that incorporate the essences of both theory and simulation (Nichols and Stich, 2003; Saxe, 2005; Goldman, 2006; Mitchell et al., 2009). Notably, Mitchell et al. (2009) proposed that children first acquire a competence of simulation and then develop a theory-based reasoning skill, and they will adopt both of these reasoning strategies depending on the demands of a particular task at hand. Recent neuroimaging findings further support such hybrid approaches, demonstrating mixed evidence for the neural mechanisms of ToM responsible for either theory-based or simulation-based reasoning (Apperly, 2008; Mahy et al., 2014).

In spite of a large body of empirical findings and recent theoretical advances in ToM research, relatively few studies have proposed computational models of ToM understanding, particularly false belief understanding (O'Laughlin and Thagard, 2000; Goodman et al., 2006; Berthiaume et al., 2013). Moreover, none deal with mixed reasoning strategies based on theory-theory and simulation theory. Therefore, it still remains unclear whether and how children can, in principle, combine both strategies into a coherent theory of mind. In this study, we present a computational model that integrates theory-based and simulation-based strategies for false belief reasoning. Our model builds on a Bayesian framework and thus provides a rational account of children's ToM. It also makes testable predictions about children's performance on false belief tasks, allowing a quantitative comparison with existing behavioral data.

We argue that a developmental ToM scale (Wellman and Liu, 2004) is of particular relevance for any computational model of false beliefs. The ToM scale consists of tasks to assess children's understanding of multiple mental state concepts. It reflects extant findings of children's ToM such that they develop an understanding of diverse desires (people can have different desires for the same thing) before developing that of diverse beliefs (people can have different opinions and beliefs about the same situation); they develop understandings of diverse beliefs and knowledge access (others can have different perspectives that prevent them from having access to the true real-world information) before developing that of false beliefs. This kind of developmental sequence has been confirmed for preschool children with diverse cultural backgrounds (Wellman and Liu, 2004; Peterson et al., 2005; Wellman et al., 2006; Toyama, 2007; Shahaeian et al., 2011; Hiller et al., 2014). From a constructivism point of view, such a sequential progression of ToM suggests that an understanding of false beliefs should emerge under the developed understandings of the mental concepts such as diverse desires, diverse beliefs, and knowledge access. Taking into account this view, we formalize a model of false belief reasoning based on a Bayesian network (Pearl, 2000; Spirtes et al., 2001) that represents causal relationships among the relevant mental concepts of others and one's own. In so doing, we show that this Bayesian network in effect provides a natural way to integrate theory-based and simulation-based strategies. We further demonstrate that our model provides a good fit to the existing ToM scale data.

## 2. Model

### 2.1. Bayesian network

A Bayesian network is a graphical model that provides a compact representation of the joint probability distribution for a set of random variables (Pearl, 2000; Spirtes et al., 2001). Its graph structure represents the causal probabilistic relationship among the variables, specifies a particular factorization of the joint probability distribution, and enables efficient computation of probability distributions of the unobserved variables, given the observed ones. Bayesian networks have been used in a wide range of fields and applications, such as computer science, engineering, statistics, medical diagnosis, and bioinformatics. Recently, they have found application in various areas of psychology, such as visual perception (Kersten et al., 2004), cognition (Griffiths et al., 2008; Jacobs and Kruschke, 2011), causal inference (Griffiths and Tenenbaum, 2005; Lu et al., 2008), and cognitive development (Gopnik et al., 2004; Gopnik and Tenenbaum, 2007; Gopnik and Wellman, 2012). Notably, Gopnik and Wellman (2012) have argued that Bayesian networks can be used for formalizing a theory-theory of cognitive development. In the following, we use this Bayesian network formalism to elaborate a model of false belief reasoning in children.

From a theory-theory perspective, Goodman et al. (2006) proposed Bayesian network models of false belief reasoning in the change-of-location task. Our Bayesian formulation closely follows their work; the differences are that we additionally take into account the idea of simulation theory and that we focus on the unexpected-contents task to model false belief reasoning. The latter is motivated by the fact that this type of task was commonly used across the ToM scale studies listed above, but has not been the subject of formal analysis. The extension of our model to the change-of-location task will be discussed later.

We note that the unexpected-contents task is divided into two stages. According to Wellman and Liu (2004), in the first stage, a child sees a familiar, closed Band-Aid box that holds inside a plastic pig toy. From the appearance of the Band-Aid box, the child first expects Band-Aids inside. Subsequently, the Band-Aid box is opened and the child sees the pig toy inside. Then, the Band-Aid box is closed again. In the second stage, there is a toy figure of a boy named Peter. The child hears that Peter has never seen inside this Band-Aid box. Then, the child answers the question: What does Peter think is in the box? Band-Aids or a pig? Thus for the child, the first stage concerns updating the belief state of the self, whereas the second stage involves reasoning about the belief state of others.

These consecutive stages can be formalized within a Bayesian network framework as follows. Consider the process of belief updating in the first stage. The initial belief about the hidden contents of the Band-Aid box (i.e., Band-Aids) comes from observing the outside of the box. Then, the updated belief (i.e., the pig toy) arises from having visual access to the inside of the box. This in turn implies that without such visual access, the initial belief wound not be updated, but instead remain in its original state.

**Figure 1:**
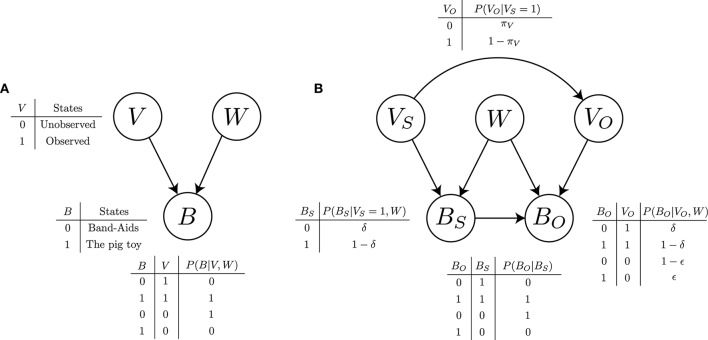
**(A)** Bayesian network describing a causal relation among the states of the world (*W*), visual access (*V*), and belief (*B*). The conditional probability table indicates that the belief updating is deterministic. **(B)** Our Bayesian network model for false belief reasoning. The *S* subscript is the abbreviation for “self;” the *O* subscript for “others.”

These causal relationships can be concisely represented with a causal graphical model or a Bayesian network, as depicted in Figure 1A. This graph has three nodes of random variables (*W*, *V*, and *B*) and two directed edges between the nodes (i.e., single arrows). *W* represents the true state of the world, that is, the Band-Aid box that holds the pig toy inside. *V* represents the binary states of visual access to the inside of the box: the contents are observed or not. *B* represents the binary states of the belief about the contents: the pig toy (true belief) or Band-Aids (false belief). The directed edges represent causal connections between these variables: each arrow points from a cause to an effect. Accordingly, this graph specifies a causal relation such that *V* and *W* are the cause of *B*. In addition, it implies a probabilistic interpretation of the causal relation in terms of the conditional probability of *B* given *V* and *W*: *P*(*B*|*V, W*). To be more specific, this graph defines the joint probability distribution over all variables *P*(*B, V, W*), and the causal structure implies a particular factorization of the joint probability distribution as *P*(*B, V, W*) = *P*(*B*|*V, W*)*P*(*V*)*P*(*W*). This Bayesian network thus denotes how belief formation proceeds in children's minds for the unexpected-contents task.

Next, consider belief reasoning in the second stage. Given the updated belief in the first stage and available information about Peter's visual access, the child makes an inference about Peter's belief about the hidden contents of the Band-Aid box. We propose that the child makes a probabilistic inference about Peter's belief using a “theory” that represents the process of belief updating in the first stage. Specifically, we propose that at the computational level (Marr, 1982), the child's belief reasoning can be formalized as probabilistic computations on a Bayesian network that represents the causal process of belief formation (Figure 1B).

To construct this Bayesian network, we make two assumptions. First, we assume that the child has two theories of belief formation, one applied to her and the other to Peter, each of which can be represented as the Bayesian network in Figure 1A. In other words, we postulate two internal models (Wolpert et al., 2003) of self and others for belief prediction. In effect, the internal model of self acts as a simulator of one's own mind. This can be viewed as a form of rule-based simulation (cf. Mitchell et al., 2009). Second, we assume that during children's early development, their internal model of self is inseparable from and affects that of others. Specifically, we hypothesize that when reasoning about Peter's belief, the child's own states of belief and visual access have an effect on those in the internal model of Peter. This hypothesis embodies a true-belief default (Leslie et al., 2004): others tend to have the same true belief as one's own, as people usually have true beliefs about everyday matters. It also follows from the “like-me” hypothesis of infant social cognitive development (Meltzoff, 2007a,b).

These two assumptions lead us to construct the Bayesian network depicted in Figure 1B. This graph has two sets of random variables: *V*_*S*_ and *B*_*S*_, representing the binary states of visual access and belief, respectively, of self; and *V*_*O*_ and *B*_*O*_ of others. Given *W*, each set of the variables can constitute a subgraph that exactly matches the graph in Figure 1A. Then, merging the two subgraphs leads to jointly representing two internal models of belief updating: one for the self and one for others. This meets the first assumption. Further, in Figure 1B, the whole graph has two extra edges between the variables of the self and others: one from *V*_*S*_ to *V*_*O*_, and the other from *B*_*S*_ to *B*_*O*_. This represents their causal connections and thereby reflects the second assumption. Furthermore, this Bayesian network can be interpreted as representing the joint probability distribution over all relevant variables, implying its factorization as *P*(*V*_*O*_, *B*_*O*_, *V*_*S*_, *B*_*S*_, *W*) = *P*(*B*_*O*_|*V*_*O*_, *B*_*S*_, *W*)*P*(*V*_*O*_|*V*_*S*_)*P*(*B*_*S*_|*V*_*S*_, *W*)*P*(*V*_*S*_)*P*(*W*).

The Bayesian network in Figure 1B thus describes our proposed theory of mind that children might use in the unexpected-contents false belief task. From now on, we refer to this Bayesian network as the ToM network. Its use allows children to perform theory-based reasoning about the belief states of others. But this reasoning does not strictly follow the standard theory-theory in which the mental states of others are detached from those of one's own. Rather, reasoning with this ToM network inevitably entails more or less simulation-based reasoning through the internal model of one's own mind. Therefore, our proposed ToM network can be viewed as a hybrid model combining both theory-based and simulation-based reasoning. In the following section, we formulate false belief reasoning as Bayesian inference with such a hybrid model. This can be done through parameterization of the ToM network to specify a conditional probability for each relevant variable.

### 2.2. False belief reasoning

First, we introduce notations to denote the states of a binary variable. For the states of visual access to the inside of the Band-Aid box, let *V*_*S*_ and *V*_*O*_ take on the value of 1 when the contents are observed and 0 otherwise. For the states of belief about the contents, let *B*_*S*_ and *B*_*O*_ take on the value of 1 for the pig toy (true belief) and 0 for Band-Aids (false belief).

Given the ToM network, reasoning about the belief state of others amounts to estimating the state of the variable *B*_*O*_ from available information about the remaining variables. For this estimation, the Bayesian approach suggests using the predicted probability *P*(*B*_*O*_|*V*_*S*_ = 1, *W*): the conditional probability of *B*_*O*_ given the observed state of *V*_*S*_ and the fixed state of *W*. Recall that the ToM network represents the joint probability distribution over all variables. Then, from this, the predicted probability can be computed by applying Bayes' rule and marginalization:

(1) $$\begin{array}{r} P\left(B_{O}|V_{S}=1,W\right)=\underset{V_{O}}{\sum }\underset{B_{S}}{\sum }P\left(B_{O}|V_{O},B_{S},W\right)P\left(V_{O}|V_{S}=1\right)\\ P\left(B_{S}|V_{S}=1,W\right) \end{array}$$

Here, the summation is taken over all possible values of *V*_*O*_ and *B*_*O*_. Hence, to formulate this predicted probability, it is necessary to specify three conditional probabilities on the right side of the Equation (1).

First consider *P*(*B*_*S*_|*V*_*S*_ = 1, *W*). This conditional probability concerns only the mental states of self and represents children's belief updating in the first stage of the unexpected-contents task. As the child comes to hold the true belief when she observes the inside of the box (i.e., *V*_*S*_ = 1), *B*_*S*_ will take on the value of 1 when her belief is just updated. We assume, however, that the child may fail to maintain the updated belief owing to accidental error, or the limited capacity of her working memory. Assuming that such failure occurs with small probability δ, we set

(2) $$\begin{array}{r} P\left(B_{S}=0|V_{S}=1,W\right)=\delta \end{array}$$

(3) $$\begin{array}{r} P\left(B_{S}=1|V_{S}=1,W\right)=1-\delta \end{array}$$

Next, consider *P*(*V*_*O*_|*V*_*S*_ = 1). This conditional probability is due to our assumption that the mental states of self have an effect on the representations of those of others. As the child hears that Peter has not observed the inside of the box, *V*_*O*_ should be 0 if she correctly identifies the state of Peter's visual access. However, our assumption states that the child may mistakenly attribute her state of mind to Peter. Assuming that this happens with probability 1 − π_*V*_, we set

(4) $$\begin{array}{r} P\left(V_{O}=0|V_{S}=1\right)=\pi_{V} \end{array}$$

(5) $$\begin{array}{r} P\left(V_{O}=1|V_{S}=1\right)=1-\pi_{V} \end{array}$$

Thus, π_*V*_ expresses the degree to which children can appreciate the states of others' visual access, or equivalently, the knowledge states of others.

Finally, consider *P*(*B*_*O*_|*V*_*O*_, *B*_*S*_, *W*). This conditional probability represents the process of others' belief updating with its dependence on the belief states of self. This dependence is again due to our assumption stated above. We assume that the child may automatically adopt her state of belief to represent Peter's own and this occurs with a probability of 1 − π_*B*_. This prompts us to decompose *P*(*B*_*O*_|*V*_*O*_, *B*_*S*_, *W*) as follows:

(6) $$\begin{array}{l} P\left(B_{O}|V_{O},B_{S},W\right)=\pi_{B}P\left(B_{O}|V_{O},W\right)+\left(1-\pi_{B}\right)P\left(B_{O}|B_{S}\right) \end{array}$$

This decomposition implies that to represent Peter's belief, the child uses *P*(*B*_*O*_|*V*_*O*_, *W*) with probability π_*B*_, or *P*(*B*_*O*_|*B*_*S*_) with probability 1 − π_*B*_. Thus, π_*B*_ expresses the degree to which children can attribute different beliefs to others.

Note that *P*(*B*_*O*_|*V*_*O*_, *W*) concerns only the mental states of others and represents the identical causal structure with *P*(*B*_*S*_|*V*_*S*_, *W*). Therefore, as Equations (2) and (3), when *V*_*O*_ = 1, we set

(7) $$\begin{array}{r} P\left(B_{O}=0|V_{O}=1,W\right)=\delta \end{array}$$

(8) $$\begin{array}{r} P\left(B_{O}=1|V_{O}=1,W\right)=1-\delta \end{array}$$

When *V*_*O*_ = 0, Peter should hold the false belief (*B*_*O*_ = 0) because he observes only the outside of the Band-Aid box. However, we assume that the child takes into account the possibility that Peter expects something other than Band-Aids inside the box. This can happen because the box is a container that can hold anything smaller than its size; in fact, it held the pig toy inside in the unexpected-contents task. Assuming that the child supposes such a misconception could occur with a small probability ϵ, we set

(9) $$\begin{array}{r} P\left(B_{O}=0|V_{O}=0,W\right)=1-\epsilon \end{array}$$

(10) $$\begin{array}{r} P\left(B_{O}=1|V_{O}=0,W\right)=\epsilon \end{array}$$

For *P*(*B*_*O*_|*B*_*S*_), we assume that it is deterministic since its probabilistic nature has already been captured with the probability 1 − π_*B*_. That is, assuming that the child's state of belief is just copied to Peter's belief, we set

(11) $$\begin{array}{r} P\left(B_{O}=0|B_{S}=0\right)=P\left(B_{O}=1|B_{S}=1\right)=1 \end{array}$$

(12) $$\begin{array}{r} P\left(B_{O}=0|B_{S}=1\right)=P\left(B_{O}=1|B_{S}=0\right)=0 \end{array}$$

By using the parameterization introduced thus far, we can derive the predicted probability *P*(*B*_*O*_|*V*_*S*_ = 1, *W*), and then our model of false belief reasoning as *P*(*B*_*O*_ = 0|*V*_*S*_ = 1, *W*). First, we substitute Equation (6) into Equation (1) to obtain:

(13) $$\begin{array}{r} P\left(B_{O}|V_{S}=1,W\right)=\pi_{B}\underset{V_{O}}{\sum }P\left(B_{O}|V_{O},W\right)P\left(V_{O}|V_{S}=1\right)\\ +\;\left(1-\pi_{B}\right)\underset{B_{S}}{\sum }P\left(B_{O}|B_{S}\right)P\left(B_{S}|V_{S}=1,W\right) \end{array}$$

This illustrates how theory-based and simulation-based strategies are combined to perform reasoning about the belief state of others. The first term means that the child first obtains an estimate of the state of Peter's visual access using her own state (*P*(*V*_*O*_|*V*_*S*_ = 1)), then feeds the estimate into the internal model of others (*P*(*B*_*O*_|*V*_*O*_, *W*)) to predict the belief state of Peter. This corresponds to a theory-based strategy. In contrast, the second term means that the child first employs the internal model of self (*P*(*B*_*S*_|*V*_*S*_ = 1, *W*)) to simulate her own belief updating and then projects the simulated state of belief onto Peter (*P*(*B*_*O*_|*B*_*S*_)). This corresponds to a simulation-based strategy. The probability π_*B*_ acts as a gate to select one of these strategies: the child performs theory-based reasoning with probability π_*B*_ and simulation-based reasoning with probability 1 − π_*B*_.

Then, by setting *B*_*O*_ = 0 and summing *V*_*O*_ and *B*_*S*_ in Equation (13), we finally obtain our model of false belief reasoning:

(14) $$\begin{array}{l} P\left(B_{O}=0|V_{S}=1,W\right)=\pi_{B}\pi_{V}\left(1-\epsilon \right)+\left(1-\pi_{B}\pi_{V}\right)\delta \end{array}$$

This is the probability that given knowledge about the true state of the world, the child estimates the belief state of Peter as a false belief. In the following, we denote this probability as π_*FB*_.

### 2.3. Relation to the theory-of-mind scale

Our model of false belief reasoning takes four probabilities as its parameters: δ, ϵ, π_*B*_, and π_*V*_. Of these, the effects of δ and ϵ are likely to be limited since they are assumed to be small, random errors. This is justified by the procedure employed in all the ToM scale studies listed above. In fact, for children's false belief responses to be scored as correct, the children were first required to respond correctly to preliminary and control questions about what is usually in a Band-Aid box (i.e., Band-Aids) and what is actually in the Band-Aid box presented (i.e., the pig toy). Thus, we can safely assume that children rarely, if ever, came up with something other than Band-Aids inside the box (i.e., small ϵ) and failed to maintain the updated belief about the contents of the box (i.e., small δ). In contrast, the remaining two probabilities, π_*B*_, and π_*V*_, play a dominant role in specifying the behavior of our model. Let us remember that π_*B*_ and π_*V*_ are introduced to quantify children's abilities to differentiate their mental states, beliefs, and visual access, respectively, from those of others. These abilities as well as false belief reasoning are, in fact, ToM skills that are to be assessed with the ToM scale (Wellman and Liu, 2004). Two relevant ToM tasks included in the scale: diverse beliefs and knowledge access. A diverse-beliefs task involves the ability to understand that others can have different beliefs about the same situation. A knowledge access task involves the ability to discern others' visual access to judge whether they are knowledgeable or ignorant. Hence, π_*B*_ corresponds to the proportion correct for the diverse-beliefs task, and π_*V*_ for the knowledge access task. Obviously, π_*FB*_ amounts to children's proportion correct for the unexpected-contents false-beliefs task.

Our model thus predicts that, when assessed with the ToM scale in terms of the proportion correct, children's false belief ability can be predicted through their abilities to understand diverse beliefs and knowledge access. Indeed, assuming that δ and ϵ are sufficiently small, we can approximate Equation (14) to obtain a simple relation: π_*FB*_ ≈ π_*B*_π_*V*_. It follows that false belief reasoning can be viewed as a multiplicative effect of understanding diverse beliefs and knowledge access. Below we will show that this simple multiplicative relation holds across a wide variety of children's ToM scale data.

## 3. Results

We illustrated the validity of our model by fitting the full model of four parameters using a Bayesian method to the children's ToM scale data for the three above-mentioned tasks. The data for each task consist of the number of children who successfully completed the task. To fit our model to the data, we take the proportion of children who were correct on each task as their proportion correct for the task. Specifically, we assume an individual child's responses to these tasks as independent Bernoulli trials with success probabilities π_*B*_ for diverse beliefs, π_*V*_ for knowledge access, and π_*FB*_ for unexpected-contents false belief. This allows us to derive the joint likelihood function of the parameters π_*B*_, π_*V*_, δ, and ϵ (note that π_*FB*_ is a function of them). In addition, similar to Goodman et al. (2006), we assume asymmetric beta priors on δ and ϵ to make their small values more likely than large ones. Then, we combine the likelihood and prior to form the posterior distribution over the parameters, from which we find their maximum a posteriori estimates (see the Appendix for details). Given these estimates, we will obtain a prediction of π_*FB*_ using Equation (14).

**Figure 2:**
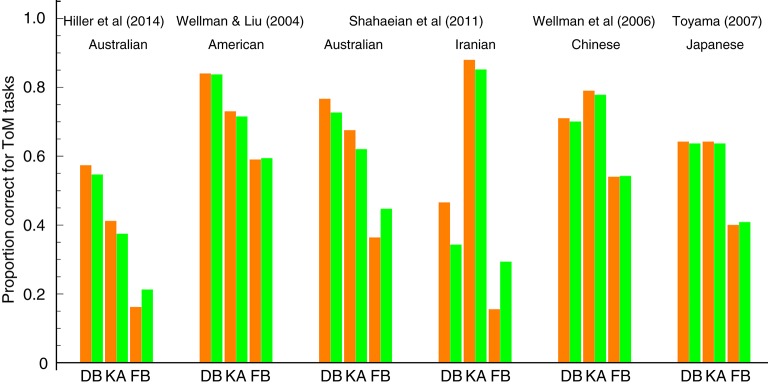
**Comparisons between our model prediction and behavioral ToM scale data for three tasks: diverse beliefs (DB), knowledge access (KA), and false beliefs (FB)**. Orange bars represent the behavioral data. Green bars represent the fits of our prediction (the estimated values of the parameters π_*B*_, π_*V*_, and π_*FB*_). The left part of the figure depicts the results for Western children (Australian and American); the right part for Asian/Middle Eastern children (Iranian, Chinese, and Japanese).

Figure 2 shows the comparison of our model prediction with the data from several ToM scale studies (Wellman and Liu, 2004; Peterson et al., 2005; Wellman et al., 2006; Toyama, 2007; Shahaeian et al., 2011; Hiller et al., 2014). These studies recruited participants from the same age group (range: 3–6 years), but differed in their choice of the children's cultural background. One exception is Hiller et al. (2014), whose focus was on a younger age group including 2-year-old children. For each study, we fitted our model to the aggregated data from all participants with varying ages.

For three ToM tasks considered in our model, the above studies revealed different orders of difficulty between cultures. Western, English-speaking children mastered the tasks in the following order: diverse beliefs, then knowledge access, and finally false beliefs. In contrast, Asian/Middle Eastern peers reversed the order between diverse beliefs and knowledge access (Note that these orders only indicate cross-sectional ToM progressions. However, Wellman et al. (2011) have recently revealed that children's longitudinal ToM progressions assessed with the ToM scale follow the same orders as obtained cross-sectionally. This validates the use of the cross-sectional data as a good approximation of the longitudinal sequences of ToM understanding for individual children). As demonstrated in Figure 2, our model is able to capture this cross-cultural contrast. It predicts that children's abilities to understand diverse beliefs and knowledge access multiplicatively contribute to their ability to understand false beliefs. Hence it further predicts that the order of difficulty between the former two tasks is irrelevant to the level of false belief understanding. Our fitting results confirmed these predictions, almost quantitatively reproducing both the ToM scale data from Western and Non-Western children.

**Figure 3:**
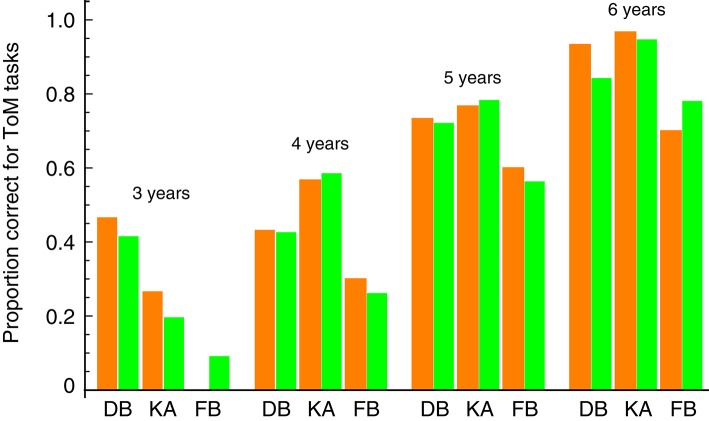
**Comparison between our model prediction and the ToM scale data of different age groups in Toyama (2007)**. DB, diverse beliefs; KA, knowledge access; FB, false beliefs. Orange bars represent the behavioral data. Green bars represent the fits of our prediction (the estimated values of the parameters π_*B*_, π_*V*_, and π_*FB*_).

Note that our model is in good agreement with the data from Hiller et al. (2014), whose focus was on a younger age group including 2-year-old children. This suggests that our model can apply to separate age groups to predict each age-related level of ToM development. We examined this possibility using ToM scale data for Japanese children (Toyama, 2007) from the only study among those listed above that reported children's ToM task performance at every age group between 3 and 6 years old. Figure 3 shows a separate fit of our model to the data for each age group. The model fits were good, and the linear correlation between the estimated parameters (i.e., π_*B*_, π_*V*_, and π_*FB*_) and the data over all age groups was 0.98. These results indicate that our model can capture the pattern of children's ToM abilities at each stage of their development.

**Figure 4:**
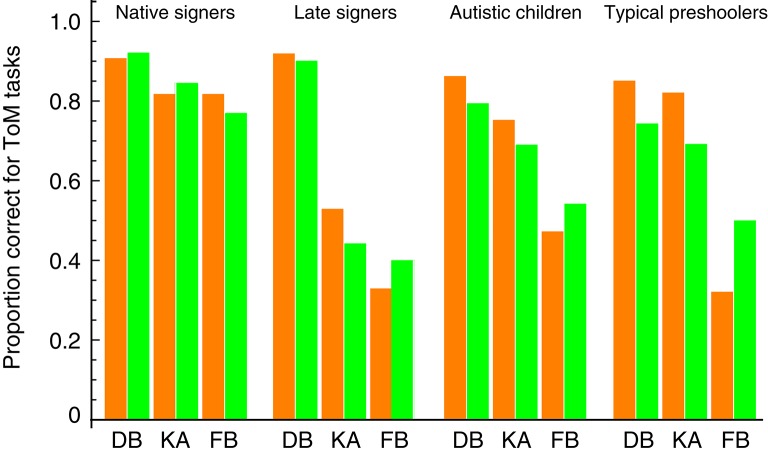
**Comparison between our model prediction and the ToM scale data of children with and without developmental delays in Peterson et al. (2005)**. DB, diverse beliefs; KA, knowledge access; FB, false beliefs. Orange bars represent the behavioral data. Green bars represent the fits of our prediction (the estimated values of the parameters π_*B*_, π_*V*_, and π_*FB*_).

Finally, we assessed whether our model can apply to children with developmental delays. Peterson et al. (2005) compared sequences of ToM development between typically developing and ToM-delayed Australian children with deafness or autism and found the same order of difficulty across all children groups, at least for three ToM tasks considered in our model. Figure 4 shows separate fits of our model to the data for four children groups: native signers (deaf children born to signing deaf parents, mean age 10.67 years), late signers (deaf children born to non-signing hearing parents, mean age 10.01 years), autistic children (mean age 9.32 years), and typical preschoolers (mean age 4.50 years). The model fits were good even for ToM-delayed children (i.e., late singers and children with autism), suggesting that their ToM abilities, albeit delayed in development, should work in the same way as native signers and typical children.

## 4. Discussion

We have formalized a Bayesian model of false belief reasoning that incorporates the internal models of self and others for belief formation. This model can be viewed as a version of theory-theory, explicitly representing a set of mental concepts and their interactions by a probabilistic causal model (Gopnik and Wellman, 2012). Critically, however, our model differs from the standard theory-theory in that it possesses a theory of one's own mind as well as that of other people's minds. Moreover, it allows simulation-based and standard theory-based strategies for reasoning about the belief states of others to be integrated. We have demonstrated that this hybrid approach can capture various aspects of ToM scale findings: cultural differences, age-wise development, and developmental delays with autism and deafness.

Our model predicts children's false belief ability as a multiplicative effect of their abilities to understand diverse beliefs and knowledge access. As shown above, in terms of success probabilities for corresponding ToM scale tasks, this prediction can be concisely expressed as: π_*FB*_ ≈ π_*B*_π_*V*_. It is important to remember that the latter two probabilities are introduced into our model to represent the degree to which children can discern their mental states, beliefs, and visual access, from those of others. In effect, the larger these probabilities, the larger π_*FB*_, and the stronger the tendency for children to recognize that others are not “like-me” in their mental states. Thus, our model predicts that developed false belief reasoning (i.e., larger π_*FB*_) should rest predominantly on the internal model of others to employ a theory-based strategy and that conversely, undeveloped false belief reasoning (i.e., smaller π_*FB*_) should be based mainly on the internal model of self to employ a simulation-based strategy. This differential weighting between the internal models of self and others enables our model to account for a wide variety of ToM scale data, capturing the variability of false belief ability observed across those behavioral studies.

Thus, our model is able to characterize children's competence in false belief reasoning at the various stages and aspects of their development. However, the model is not itself, in its current formulation, a model for ToM acquisition. Nevertheless, it provides preliminary evidence regarding how ToM development proceeds in childhood. The key point is again the multiplicative relation: π_*FB*_ ≈ π_*B*_π_*V*_. The relation states that a larger π_*FB*_ requires both π_*B*_ and π_*V*_ to be much larger simultaneously. Therefore, it implies that children's earlier understanding of diverse beliefs and knowledge access is a prerequisite for promoting their later false belief understanding. This naturally corresponds to a constructivist account of ToM development. Specifically, our model follows an approach of rational constructivism in cognitive development (Xu and Kushnir, 2013), as it builds on a Bayesian framework to make rational inferences. Hence, our model gives a formal constructivist interpretation of the sequential progression of ToM understandings assessed with a ToM scale.

Regarding the process of ToM development, our model makes another constructivist prediction with the multiplicative relation: π_*FB*_ ≈ π_*B*_π_*V*_. A key observation is that the relation is bilinear: π_*FB*_ is linear in π_*B*_ when π_*V*_ is fixed and vice versa. This means that a fixed level of π_*B*_ or π_*V*_ affects the slope in the linear function of the other. Hence, provided that either π_*B*_ or π_*V*_ is fixed to a certain level and the other increases monotonically, a higher fixed level will result in a faster increase in the level of π_*FB*_. This leads us to predict that children's initial level of understanding of diverse beliefs or knowledge access determines how fast their later understanding of false beliefs progresses over the course of ToM development. This prediction is qualitatively consistent with a recent microgenetic study by Rhodes and Wellman (2013). They demonstrated that children who had a well-developed understanding of knowledge access reliably developed an understanding of false beliefs following repeated observations of other people acting on false beliefs, whereas children who had an undeveloped understanding of knowledge access did not. Our model further predicts that children's level of diverse belief understanding also constrains their development of false belief understanding. This is due to the fact that the roles of π_*B*_ and π_*V*_ are interchangeable in our model. Pursuing this idea in a future empirical study would be worthwhile to test the prediction with microgenetic methods.

We should finally note that our Bayesian model of false beliefs, however successful, is only applicable to the unexpected-contents task, and not to the change-of-location task. To formalize false belief reasoning in the latter task, we need a related but different theory, or causal structure, to represent relevant mental state concepts. Specifically, the change-of-location task involves an extra representation of other people's actions (i.e., where to look for an object). In addition, the state of their actions depends not only on that of their beliefs (where the object is located), but also on the state of their desires (whether they want the object).

Representing this causal structure as Bayesian networks, Goodman et al. (2006) proposed two models of false beliefs: a copy theorist (CT) model and a perspective theorist (PT) model. The CT model assumes that others' beliefs depend only on the real state of the world. In contrast, the PT model assumes that they further depend on others' visual access to the world. Then it follows that the CT model is able to represent true beliefs, but is too simple to represent false beliefs, whereas the PT model is complex enough to represent both true and false beliefs. Goodman et al. modeled the development of the false belief ability as a rational transition from the CT model to the PT model: one of these models is selected for false belief reasoning according to their corresponding posterior model probabilities.

Thus, for the change-of-location task, a similar computational explanation has been advanced to understand false belief reasoning. However, similar to most behavioral ToM studies, Goodman et al. (2006) have focused on false beliefs *per se*, without taking into account extended developmental progressions of ToM leading up to false belief understanding. Therefore, we argue that, by extending our Bayesian model to accommodate the change-of-location task, it will have more explanatory power in understanding children's developing ToM abilities. Such extension is rather straightforward. It simply uses Goodman et al.'s PT model (Bayesian network) as a building block for the internal models of self and others' minds. Causal connections are then added between variables of the two internal models. The relevant variables include the state of desire as well as those of belief and visual access. This extended model, in principle, allows for Bayesian inference of other people's action goal on the change-of-location task. Importantly, it can also cope with the unexpected-contents task since it reduces to our current formulation when irrelevant variables, in this case desire and action, are marginalized out. Furthermore, within the extended model, a causal influence between the desire states of self and others can be assessed with another task included in the ToM scale: a diverse-desires task. We are currently formalizing and validating the extended model, trying to fit it to ToM scale data including the false belief ability for the change-of-location task (Wellman and Liu, 2004; Shahaeian et al., 2014). Extending our model would thus make better use of ToM scale data to contribute to a more in-depth understanding of developmental progressions of ToM abilities.

## Author contributions

NA and TI designed the study and developed the model. NA performed the model fitting to behavioral data, and prepared the manuscript. TI edited the manuscript. NA and TI discussed the results and implications of this work.

### Conflict of interest statement

The authors declare that the research was conducted in the absence of any commercial or financial relationships that could be construed as a potential conflict of interest.

## Appendix

### Fitting the model of false belief reasoning to ToM scale data

We adopted a Bayesian method for estimating the parameters π_*B*_, π_*V*_, δ, and ϵ to fit Equation (14) to ToM scale data *D* for three tasks: diverse beliefs, knowledge access, and unexpected-contents false beliefs. This can be done through maximizing the posterior probability distribution for the parameters:

(A1) $$\begin{array}{l} P\left(\pi_{B},\pi_{V},\delta ,\epsilon |D\right)=\frac{P\left(D|\pi_{B},\pi_{V},\delta ,\epsilon \right)P\left(\pi_{B},\pi_{V},\delta ,\epsilon \right)}{P\left(D\right)} \end{array}$$

where *P*(*D*|π_*B*_, π_*V*_, δ, ϵ) is the likelihood function of the data and *P*(π_*B*_, π_*V*_, δ, ϵ) is the prior probability distribution for the parameters. *P*(*D*) is a normalization constant. This is due to Bayes' rule. We assume an individual child's responses to the three tasks as independent Bernoulli trials with success probabilities π_*B*_ for diverse beliefs, π_*V*_ for knowledge access, and π_*FB*_ for unexpected-contents false beliefs. We can then represent the number of children passing each task using the sum of the Bernoulli trials with the values of 1 for success and 0 for failure. This sum has a binomial distribution, giving the likelihood function of the data for each task. The joint likelihood function of the entire data set is the product of three likelihood functions for each task and is given by:

(A2) $$\begin{array}{l} L(\pi_{B},\pi_{V},\pi_{FB};D){=}_{n}{\text{C}}_{r_{B}}{\pi_{B}}^{r_{B}}{\left(1-\pi_{B}\right)}^{n-r_{B}}\cdot_{n}{\text{C}}_{r_{V}}{\pi_{V}}^{r_{V}}\\ \;{\left(1-\pi_{V}\right)}^{n-r_{V}}\cdot_{n}{\text{C}}_{r_{FB}}{\pi_{FB}}^{r_{FB}}{\left(1-\pi_{FB}\right)}^{n-r_{FB}} \end{array}$$

where *n* is the number of participating children and *r*_*B*_, *r*_*V*_ and *r*_*FB*_ are the numbers of children passing the corresponding tasks. The likelihood function *P*(*D*|π_*B*_, π_*V*_, δ, ϵ) is then obtained by substituting Equation (14) into π_*FB*_. For the prior probability distribution *P*(π_*B*_, π_*V*_, δ, ϵ), we assume uniform priors on π_*B*_ and π_*V*_, and asymmetric beta priors on δ and ϵ with a beta distribution: *Beta*(2, 48) (mode 0.02; mean 0.04; variance 0.00075). Thus, the prior probability reduces to *P*(δ)*P*(ϵ) = *Beta*(2, 48)·*Beta*(2, 48).

As shown above, the posterior distribution is proportional to the product of the likelihood and the prior distribution. For convenience, we numerically maximized the logarithm of the product with respect to the parameters to obtain their maximum a posteriori estimates. We did this using Mathematica's built-in NMaximize function with the constraint that each parameter takes values between zero and one (i.e., all parameters should be probabilities). Substituting these estimates into Equation (14), we obtained a prediction of π_*FB*_.
